# Impaired Left Atrial Strain as an Early Marker of Cardiac Involvement in Type 2 Diabetes Mellitus: A Cross-Sectional Study

**DOI:** 10.3390/jcdd12090369

**Published:** 2025-09-19

**Authors:** Laura-Cătălina Benchea, Larisa Anghel, Nicoleta Dubei, Răzvan-Liviu Zanfirescu, Gavril-Silviu Bîrgoan, Radu Andy Sascău, Cristian Stătescu

**Affiliations:** 1 Internal Medicine Department, “Grigore T. Popa” University of Medicine and Pharmacy, 700503 Iași, Romania; benchea.lauracatalina@gmail.com (L.-C.B.); nicoletadubei@yahoo.com (N.D.); silviubirgoan@gmail.com (G.-S.B.); radu.sascau@umfiasi.ro (R.A.S.); cristian.statescu@umfiasi.ro (C.S.); 2 Cardiology Department, Cardiovascular Diseases Institute “Prof. Dr. George I. M. Georgescu”, 700503 Iași, Romania; zanfirescu_razvan-liviu@d.umfiasi.ro; 3 Physiology Department, “Grigore T. Popa” University of Medicine and Pharmacy, 700503 Iași, Romania

**Keywords:** left atrial strain, diabetic cardiomyopathy, cardiovascular risk stratification, diabetes mellitus

## Abstract

**Background:** Diabetic cardiomyopathy is a major contributor to cardiovascular morbidity, often progressing silently before overt heart failure. Left atrial (LA) strain, assessed via speckle-tracking echocardiography, could serve as an early indicator of subclinical myocardial dysfunction in patients with type 2 diabetes mellitus (T2DM). **Objectives:** The objectives of this study were to evaluate LA strain parameters in patients with T2DM versus non-diabetic controls and investigate their association with glycemic control and diabetes duration. **Methods:** This cross-sectional study, designed according to STROBE reporting guidelines, included 47 participants (25 with T2DM and 22 controls) undergoing comprehensive echocardiographic and biochemical evaluation. LA reservoir (LASr), conduit (LAScd), and booster-pump (LASbp) strain values were measured. Associations with glycosylated hemoglobin (HbA1c) and diabetes duration were assessed via multivariate analysis. ROC curves were used to evaluate predictive performance. **Results:** Diabetic patients had significantly lower LASr (20.4 ± 7.25% vs. 26.7 ± 8.0%, *p* = 0.007), LAScd (−10.9 ± 5.4% vs. −15.6 ± 6.5%, *p* = 0.010), and LASbp (−9.9 ± 4.2% vs. −12.9 ± 5.0%, *p* = 0.034). LASr and LAScd remained independent predictors in multivariate models. ROC analysis showed good discrimination (AUC: LAScd = 0.78; LASr = 0.73). **Conclusions:** This study demonstrates that LASr and LAScd are independently associated with type 2 diabetes mellitus and can reliably identify subclinical atrial dysfunction before the onset of structural or symptomatic heart disease.

## 1. Introduction

Diabetes mellitus (DM) represents a significant health burden, with approximately 828 million cases reported in 2022 and projections surpassing 1.3 billion by 2050 [1,2,3]. In Romania, the PREDATORR study reported a prevalence of 11.6% for diabetes and 16.5% for pre-diabetes in adults aged 20–79 years [4]. The high morbidity and mortality associated with DM are largely due to its chronic complications, particularly cardiovascular disease (CVD) [5,6,7,8,9].

Diabetic cardiomyopathy is defined as myocardial dysfunction that develops independently of coronary artery disease, hypertension, or notable valvular abnormalities [10]. It typically begins with asymptomatic diastolic dysfunction and preserved ejection fraction, progressing to combined diastolic and systolic impairment, and ultimately to overt heart failure [11].

Although much of the research on diabetic heart disease has concentrated on left ventricular (LV) changes, increasing evidence shows that type 2 diabetes mellitus (T2DM) also affects the structure and function of the left atrium (LA) [10,11]. The LA plays a key role in facilitating LV filling and maintaining cardiac output, and early dysfunction may carry important prognostic implications.

Previous studies have documented morphological alterations in the LA among diabetic patients, including significantly larger atrial volumes compared with non-diabetic controls [12,13]. These changes, detectable even in the absence of overt cardiovascular disease, suggest early atrial remodeling. More recently, speckle-tracking echocardiography has enabled the detection of functional impairment through LA strain analysis. Lower values in reservoir, conduit, and booster-pump strain—indicators of impaired atrial compliance and contractile performance—are associated with increased likelihood of atrial fibrillation, stroke, heart failure–related admissions, and cardiovascular mortality [14,15,16].

Furthermore, evidence suggests that glycemic dysregulation may contribute to both structural remodeling and electrical instability of the atrium, potentially increasing the risk of arrhythmias [17]. However, the extent and pattern of LA strain impairment in well-characterized T2DM patients without coexisting cardiovascular disease, and its relationship with clinical and biochemical parameters, remain insufficiently studied.

The present study addresses this gap by comparing LA strain parameters between T2DM patients and age-matched non-diabetic controls and by examining their associations with markers of glycemic status. Such insights could help identify subclinical cardiac involvement at an earlier stage, improving risk stratification and informing preventive strategies.

## 2. Materials and Methods

### 2.1. Study Design

We conducted an observational, single-center cohort study at the Cardiovascular Disease Institute “Prof. Dr. George I.M. Georgescu” in Iași, Romania, designed and reported in accordance with the STROBE (Strengthening the Reporting of Observational Studies in Epidemiology) statement (Supplementary Materials Table S1). The aim of this study was to evaluate left atrial function using speckle-tracking echocardiography in patients with T2DM compared to non-diabetic controls. Data collection was performed between December 2024 and May 2025. Eligible participants were identified through electronic health records and direct referrals by treating physicians. The study protocol was approved by the Ethics Committee of “Grigore T. Popa” University of Medicine and Pharmacy of Iași on 8 July 2024 (approval number 464/2024). All participants provided written informed consent before enrollment.

### 2.2. Study Population

The diabetic group included 25 adult patients (age > 28 years) with a confirmed diagnosis of T2DM based on the 2023 European Society of Cardiology (ESC) guidelines for the management of cardiovascular disease in patients with diabetes. Eligible participants met at least one diagnostic criterion for T2DM: fasting plasma glucose ≥ 7.0 mmol/L (126 mg/dL), HbA1c ≥ 48 mmol/mol (6.5%), or 2 h plasma glucose ≥ 11.1 mmol/L (200 mg/dL) after an oral glucose tolerance test, along with symptoms of hyperglycemia [18].

Exclusion criteria were applied uniformly to both study groups and included any history of cardiovascular pathology—such as coronary artery disease, valvular dysfunction, cardiomyopathies, arrhythmias, or heart failure—as well as congenital heart disease, significant pulmonary disorders, pregnancy, lactation, or inadequate echocardiographic image quality. To ensure the absence of coronary artery disease, all participants underwent evaluation with coronary computed tomography angiography (CCTA).

The control group comprised 22 age-matched individuals without diabetes, recruited during routine clinical evaluations. All had no prior diagnosis of cardiovascular or pulmonary disease and met the same exclusion criteria as the diabetic cohort, including confirmation of the absence of coronary artery disease via CCTA.

### 2.3. Clinical Data Collection

At baseline, all participants underwent a detailed clinical evaluation. Recorded parameters included age, sex, height, weight, body mass index (BMI), body surface area (BSA, calculated via the Du Bois formula) [18], duration of diabetes, antidiabetic therapy, smoking status, and alcohol consumption. Blood pressure was measured in accordance with ESC hypertension guidelines [19]. Cardiovascular risk was estimated using the SCORE2-Diabetes algorithm [8].

Fasting venous blood samples were collected in the morning to assess the following biochemical markers: glucose (mg/dL), HbA1c (%), total cholesterol (mg/dL), low-density lipoprotein (LDL) cholesterol (mg/dL), high-density lipoprotein (HDL) cholesterol (mg/dL), triglycerides (mg/dL), creatinine clearance (mL/min/1.73 m^2^), and N-terminal proB-type natriuretic peptide (NT-proBNP) (pg/mL). All participants underwent comprehensive transthoracic echocardiography using a commercially available ultrasound system. Standard two-dimensional (2D) and Doppler parameters were recorded according to current ASE/EACVI guidelines. LA strain analysis was performed using speckle-tracking echocardiography in the apical four-chamber view, with reservoir, conduit, and contraction strain components measured for group comparison. Analyses were performed offline by two experienced echocardiographers blinded to patient group allocation. Although all analyses were conducted by two experienced echocardiographers blinded to group allocation—thereby ensuring consistency and minimizing interobserver bias—all speckle-tracking strain measurements were performed using software from a single vendor (GE EchoPAC PC version 204; GE Vingmed Ultrasound, Horten, Norway). This approach provided internal standardization; however, it may limit the external reproducibility of the results across different echocardiographic platforms.

### 2.4. Statistical Analysis

Statistical analyses were carried out using IBM SPSS Statistics version 23.0 (IBM Corp., Armonk, NY, USA). Descriptive statistics were used to characterize the study population. For continuous variables, results are presented as mean values ± standard deviations (SD), while categorical variables are expressed as absolute frequencies and percentages. The Chi-square test was used to evaluate associations between categorical variables. Comparisons between the two groups (T2DM vs. controls) were conducted using Student’s *t*-test for normally distributed data or the Mann–Whitney U test for non-normally distributed variables. Chi-square or Fisher’s exact tests were used for categorical data, as appropriate.

The associations between left atrial strain parameters and continuous clinical or biochemical variables were analyzed using Pearson’s or Spearman’s correlation coefficients, depending on data normality. To identify independent predictors of impaired atrial strain, simple and multiple linear regression models were applied.

This study was observational; thus, a formal sample size calculation was not performed prior to enrollment. However, to evaluate whether the final sample size was sufficient to detect statistically meaningful differences in left atrial function, we conducted a post hoc power analysis using G*Power 3.1.9.2 software.

Additionally, Receiver Operating Characteristic (ROC) curve analysis was performed to assess the diagnostic performance of left atrial strain parameters in distinguishing diabetic patients at risk for early cardiac involvement. The area under the curve (AUC) was used to determine accuracy, and optimal cut-off values were reported where relevant. Statistical significance was set at *p* < 0.05, and values of *p* < 0.001 were considered highly significant.

## 3. Results

### 3.1. Baseline Clinical Characteristics

A total of 47 patients were included in this study: 25 with type 2 diabetes mellitus and 22 age-matched controls without diabetes. Diabetic patients were significantly older (60.96 ± 9.80 vs. 53.77 ± 13.66 years, *p* = 0.047) and had a higher prevalence of smoking (36% vs. 0.04%, *p* = 0.001), alcohol use (28% vs. 0.04%, *p* = 0.002), hypertension (92% vs. 64%, *p* = 0.022), and dyslipidemia (100% vs. 68%, *p* = 0.005) compared to controls. In our cohort, 9 patients (19.1%) with T2DM were receiving SGLT2 inhibitors, and 2 patients (4.3%) were treated with GLP-1 receptor agonists, while none of the controls were on these therapies. Given the small number of users, subgroup analyses were not feasible; however, their potential impact on cardiac structure and function is acknowledged as a study limitation. Systolic blood pressure was significantly higher in the diabetic group (152.8 ± 21.69 vs. 136.2 ± 12.58 mmHg, *p* = 0.002), while creatinine clearance was lower (81.0 ± 15.69 vs. 91.5 ± 18.78 mL/min/1.73 m^2^, *p* = 0.045). There were no significant differences in NT-proBNP or lipid profiles (Table 1).

### 3.2. Echocardiographic Parameters

No significant differences were observed in standard left ventricular measures, including left ventricular ejection fraction (LVEF), left ventricular end-diastolic volume (LVEDV), and E/A ratio. However, diabetic patients showed a trend toward impaired global longitudinal strain (GLS) of the left ventricle (−15.18 ± 3.66% vs. −16.89 ± 4.34%, *p* = 0.156) (Table 2).

**Figure 1:**
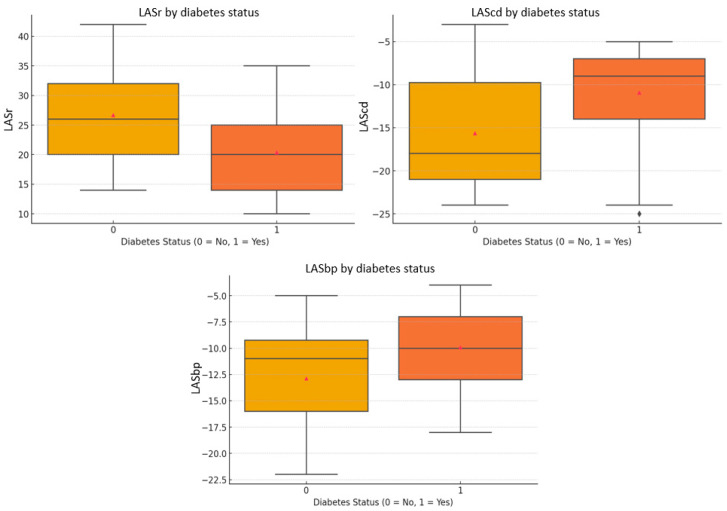
Comparison of left atrial strain values between diabetic and non-diabetic participants. Box plots display LASr, LAScd, and LASbp according to diabetes status. Group 0 (non-diabetic controls) is shown in yellow, and Group 1 (patients with T2DM) is shown in orange, consistently across all panels. LAScd and LASbp values are conventionally expressed as negative percentages; in this context, less negative values reflect impaired atrial deformation. Lower absolute strain values across all phases indicate reduced left atrial function, consistent with early subclinical myocardial involvement in diabetic cardiomyopathy. LASbp, left atrial strain at booster-pump phase; LAScd, left atrial strain at conduit; LASr, left atrial strain at reservoir.

Regarding left atrial function, diabetic patients had significantly lower reservoir strain (LASr: 20.40 ± 7.25% vs. 26.68 ± 8.00%, *p* = 0.007), conduit strain (LAScd: −10.92 ± 5.42% vs. −15.64 ± 6.46%, *p* = 0.010), and booster-pump strain (LASbp: −9.92 ± 4.20% vs. −12.87 ± 4.97%, *p* = 0.034), consistent with impaired atrial compliance and contractility (Figure 1).

The functional significance of each left atrial strain phase is as follows: LASr (reservoir strain) reflects atrial compliance and blood storage during ventricular systole (normal > 23%); LAScd (conduit strain) represents passive atrial emptying during early diastole and is sensitive to left ventricular relaxation (normal < −13%); LASbp (booster-pump strain) reflects active atrial contraction in late diastole (normal < −10%) [20,21]. In our cohort, all three phases were reduced in the diabetic group, suggesting early, diffuse impairment of atrial mechanics before structural changes become apparent.

### 3.3. Importance of LAS Parameters in Predicting Diabetic Cardiomyopathy

To evaluate independent predictors of reduced left atrial strain in diabetic patients, we conducted multiple linear regression analyses with LASr, LAScd, and LASbp as dependent variables. Duration of diabetes (years) and HbA1c (%) were included as continuous independent variables, with additional adjustment for age and SBP. Although HbA1c showed a trend toward association (OR = 1.18), it did not reach statistical significance in this model (*p* = 0.740). Neither the duration of diabetes nor SBP was an independent predictor in this sample. However, the observed trend suggested a potential relationship between worsening glycemic control and reduced atrial compliance, which warrants further investigation in larger cohorts.

In the univariate analysis, all three strain parameters were significantly associated with diabetes status. Lower LASr values were associated with increased odds of diabetes (OR = 0.90, 95% CI: 0.82–0.99, *p* = 0.027), while higher (less negative) LAScd (OR = 1.23, 95% CI: 1.07–1.41, *p* = 0.004) and LASbp values (OR = 1.16, 95% CI: 1.01–1.34, *p* = 0.038) were also predictive.

After adjusting for age, hypertension, and dyslipidemia, diabetes status remained independently associated with reduced LASr (β = −5.42%, 95% CI: −8.17 to −2.68, *p* < 0.001) and LAScd (β = −3.96%, 95% CI: −6.45 to −1.47, *p* = 0.002). The associations persisted despite the higher prevalence of these cardiovascular risk factors in the diabetic group.

To explore this further, we performed a post hoc subgroup analysis within the diabetic cohort, stratifying patients into two categories based on glycemic control: well-controlled (HbA1c < 7%; n = 14) and poorly controlled (HbA1c ≥ 7%; n = 11). Although statistical significance was not reached due to the small sample size, patients with HbA1c ≥ 7% had numerically lower LASr (19.1 ± 6.8% vs. 21.4 ± 7.7%) and LAScd (−10.1 ± 5.2% vs. −11.6 ± 5.8%) compared to those with better glycemic control. Additionally, correlation analysis between HbA1c and LA strain values across all diabetic patients showed a weak but negative correlation with LASr (r = −0.19, *p* = 0.36) and LAScd (r = −0.24, *p* = 0.24), though these did not reach significance.

**Figure 2:**
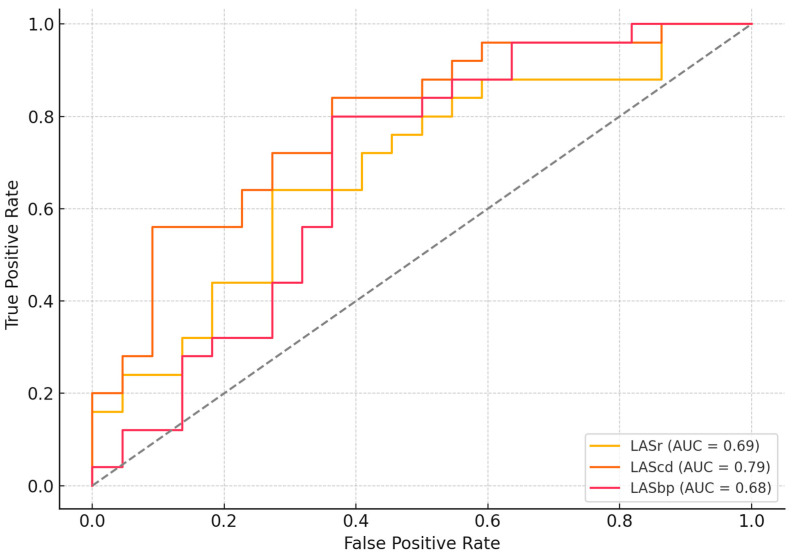
ROC curves for left atrial strain parameters (LASr, LAScd, LASbp) in predicting diabetic cardiomyopathy. LASbp, left atrial strain at booster-pump phase; LAScd, left atrial strain at conduit; LASr, left atrial strain at reservoir.

Receiver operating characteristic (ROC) curve analysis was performed to determine optimal cut-off values for discriminating between diabetic and non-diabetic participants. For LASr, the best threshold was −17%, yielding a sensitivity of 44% and specificity of 91% (AUC = 0.69). LAScd demonstrated the highest sensitivity, with an optimal cut-off of −16% (sensitivity = 92%, specificity = 59%, AUC = 0.79). For LASbp, the cut-off of −15% provided a sensitivity of 88% and specificity of 36% (AUC = 0.68). These values indicate that LAScd may be the most sensitive phase for detecting early left atrial dysfunction in patients with T2DM, while LASr offers greater specificity (Figure 2).

## 4. Discussion

This study provides new insights into the subclinical cardiac alterations associated with type 2 diabetes mellitus, emphasizing the role of advanced echocardiographic techniques—particularly left atrial strain analysis—in detecting early myocardial dysfunction. Although traditional measures such as left ventricular ejection fraction, LV volumes, and diastolic indices (E/A, E/E′) did not differ significantly between diabetic and non-diabetic patients, strain imaging revealed important distinctions, especially at the level of the left atrium. Although this study was cross-sectional and primarily exploratory, we retrospectively assessed whether the sample size was sufficient to support the statistical validity of our findings. Based on the observed difference in left atrial strain between patients with type 2 diabetes mellitus and controls, the effect size was calculated to be approximately 0.84, indicating a large effect. For the available group sizes (n = 25 and n = 22), the resulting achieved power was approximately 88%, exceeding the conventional threshold of 80% for statistical significance at a two-tailed alpha level of 0.05. The most striking findings relate to impaired left atrial strain across all phases—reservoir, conduit, and booster pump—suggesting that diabetes affects atrial mechanics early, even in the absence of overt cardiovascular disease. Our analysis demonstrates that left atrial strain parameters, particularly LAScd and LASr, are valuable tools for identifying early atrial dysfunction in diabetic patients, suggesting reduced passive emptying of the left atrium during early diastole, respectively, impaired atrial compliance and storage function during ventricular systole. Booster-pump strain, which reflects atrial contractile function during late diastole, was similarly diminished among diabetics. Importantly, the observed atrial dysfunction was present despite preserved LVEF and normal left atrial volumes, suggesting that LA strain parameters are more sensitive than conventional volumetric indices for detecting early myocardial involvement in diabetes. This pattern aligns with the concept of diabetic cardiomyopathy, in which chronic metabolic stress leads to impaired atrial compliance and contractility. Although LV GLS did not differ significantly between groups (*p* = 0.156), the lower values in diabetic patients (−15.18% vs. −16.89%) point toward early subclinical systolic dysfunction. Together, these findings support the staging hypothesis that atrial dysfunction and diastolic abnormalities precede overt ventricular systolic impairment, and highlight the potential value of combining GLS with LA strain in the early risk stratification of patients with diabetes.

In our study, a reduction of approximately 6.3% in LASr and 4.7% in LAScd in diabetic patients compared to controls reflects impaired reservoir and conduit function of the left atrium, which are early indicators of reduced atrial compliance and increased myocardial stiffness. These changes occur prior to overt structural remodeling or declines in ejection fraction. Prior research has shown that relatively small declines in LA strain, particularly LASr values under about 20%, are linked to higher rates of atrial fibrillation, worsening diastolic function, and hospitalizations for heart failure, especially among diabetic individuals [14,17]. Thus, the magnitude of strain impairment observed in our cohort may reflect a subclinical stage of diabetic cardiomyopathy, where early myocardial involvement is already present despite preserved LVEF.

By identifying these changes early, clinicians may be able to initiate more intensive risk factor control (e.g., tighter glycemic targets, cardioprotective therapies) to prevent further myocardial deterioration. We believe this strengthens the case for incorporating LA strain analysis into routine cardiovascular assessment in diabetic patients, even when conventional imaging appears normal.

The strong predictive performance of LAScd, reflected by an AUC of 0.79, suggests that this marker may be especially sensitive to subclinical myocardial changes driven by chronic metabolic stress and diastolic dysfunction. The independent significance of LASr and LAScd in multivariate analysis underscores their potential as early echocardiographic biomarkers for detecting subclinical atrial dysfunction in patients with T2DM, preceding measurable reductions in left ventricular ejection fraction or the onset of overt heart failure. Although LV global longitudinal strain did not differ significantly between groups, a trend toward impairment—alongside its strong correlation with glycemic control—suggests that left ventricular dysfunction in T2DM develops progressively and is influenced by metabolic status. Future prospective studies are warranted to explore their utility in risk stratification and monitoring therapeutic response in this population.

We acknowledge that age, hypertension, and dyslipidemia were more prevalent in the diabetic cohort, representing potential confounders. However, the independent association between diabetes and impaired LA strain persisted even after adjusting for age, hypertension, and dyslipidemia, variables that were more prevalent in the diabetic group. This strengthens the evidence that the observed atrial dysfunction is not solely explained by coexisting cardiovascular risk factors, but may reflect intrinsic myocardial involvement related to the diabetic state.

Another important result of our study is that patients with suboptimal glycemic control (HbA1c ≥ 7%) had numerically lower LASr and LAScd values compared to those with better control, although HbA1c was not identified as an independent predictor of left atrial strain in multivariate analysis. This trend, while not statistically significant, suggests a possible link between sustained hyperglycemia and early atrial functional impairment. These observations support the hypothesis that metabolic disturbances may contribute to subtle myocardial changes in diabetic patients, even before overt structural abnormalities become evident.

Previous studies have shown that LA strain is a reliable surrogate for left ventricular diastolic function and atrial mechanical performance [14,15]. Our data extend these findings by showing that diabetes independently affects all three LA functional phases, highlighting the diffuse impact of chronic hyperglycemia, oxidative stress, and myocardial fibrosis on atrial tissue mechanics. LASr, in particular, was significantly reduced in diabetics, echoing findings by Cameli et al. [14] and Bytyci et al. [15], who linked impaired LAS to worse cardiovascular outcomes in diabetes.

While left ventricular global longitudinal strain showed a trend toward impairment in diabetic patients, this difference did not reach statistical significance. Nonetheless, this observation aligns with the concept that LV systolic dysfunction in diabetes develops progressively and may be influenced by metabolic status. Prior research has demonstrated that impaired GLS in diabetic patients correlates with poor glycemic control and predicts adverse cardiovascular outcomes, even in those with preserved EF [22,23]. The numerically lower GLS values in our diabetic cohort, combined with significantly reduced LAS, support a staging hypothesis wherein atrial impairment precedes overt ventricular systolic dysfunction.

Although atrial fibrillation was not specifically evaluated in our study population, impaired left atrial strain is widely recognized as a marker of pathological remodeling and increased chamber stiffness—key mechanisms in AF pathogenesis. Evidence from prospective cohorts indicates that reductions in LA reservoir and conduit strain are independently associated with a higher incidence of new-onset AF in diverse patient groups. In the setting of type 2 diabetes mellitus, such early functional impairments may promote an arrhythmogenic substrate through structural remodeling, altered compliance, and increased fibrosis. Recent work has further shown that diabetic patients with paroxysmal AF demonstrate more severe reductions in LA strain and greater stiffness indices compared to diabetic individuals in sinus rhythm [24]. These observations support the concept that LA strain assessment may provide incremental value in identifying T2DM patients at elevated AF risk, complementing its role in the detection of subclinical atrial dysfunction.

The early detection of diabetic cardiomyopathy remains a clinical challenge, particularly in asymptomatic patients. Our data suggest that left atrial strain—especially LASr and LAScd—could serve as early and non-invasive imaging biomarkers for subclinical cardiac involvement in T2DM. These parameters may be particularly useful in guiding risk stratification and monitoring responses to interventions aimed at glycemic control and cardiovascular protection. These findings also demonstrate that diabetes mellitus is associated with a global deterioration of left atrial function, affecting not only passive compliance but also active atrial contraction. The reductions in all three strain phases support the hypothesis of diabetic cardiomyopathy, even in the absence of overt structural changes.

## 5. Limitations

This analysis represents an initial, observational component of a broader prospective study designed to re-evaluate participants at 6 and 12 months. As such, the present findings provide only a baseline snapshot and cannot capture the temporal evolution or prognostic significance of left atrial strain impairment. The small, single-center sample may limit external validity and reduce statistical power for subgroup analyses. All strain measurements were obtained using 2D speckle-tracking from a single vendor, ensuring internal consistency but limiting cross-platform reproducibility. Although major cardiovascular conditions were excluded and key confounders adjusted for, residual influences from unmeasured factors—such as coronary microvascular dysfunction, metabolic control, or specific drug therapies—cannot be ruled out. Outcome data are not yet available and will be addressed in the longitudinal phases.

## 6. Conclusions

In this observational, hypothesis-generating baseline analysis of an ongoing longitudinal study, we found that left atrial reservoir and conduit strain are significantly reduced in patients with type 2 diabetes mellitus, despite preserved left ventricular ejection fraction and the absence of overt structural heart disease. Early changes in LA function likely signal underlying myocardial involvement, reinforcing the potential of LA strain as a sensitive imaging marker for detecting diabetic cardiomyopathy in its subclinical stage. While our cross-sectional results cannot establish causality, the planned 6- and 12-month follow-ups will allow assessment of temporal changes, prognostic relevance, and the potential impact of targeted interventions on atrial function and clinical outcomes.

## Figures and Tables

**Table 1:** Patient characteristics at baseline.

Variable	Patients with DM (n = 25 Patients)	Patients Without DM (n = 22 Patients)	*p*-Value
**Demographic characteristics and cardiovascular risk factors**			
**Age, mean ± SD**	60.96 ± 9.80	53.77 ± 13.66	0.047
**Female, n (%)**	10 (40%)	11 (50%)	0.506
**Smoking, n (%)**	9 (36%)	1 (4.5%)	**0.001**
**Alcohol user, n (%)**	7 (28%)	1 (4.5%)	**0.002**
**Hypertension, n (%)**	23 (92%)	14 (63.6%)	**0.022**
**Dyslipidemia, n (%)**	25 (100%)	15 (68.2%)	**0.005**
**Admission hemodynamics**			
**BSA (m^2^), mean ± SD**	1.99 ± 0.24	1.98 ± 0.19	0.875
**Heart rate (bpm), mean ± SD**	77.92 ± 12.46	73.82 ± 11.85	0.253
**SBP (mmHg), mean ± SD**	152.80 ± 21.69	136.18 ± 12.58	**0.002**
**DBP (mmHg), mean ± SD**	86.72 ± 11.78	84.18 ± 9.15	0.411
**Biological profile**			
**NT-proBNP (pg/L), mean ± SD**	140.75 ± 81.66	93.19 ± 50.74	0.219
**Glycosylated hemoglobin (%), mean ± SD**	6.78 ± 1.10	4.97 ± 0.42	0.125
**Total cholesterol (mg/dL), mean ± SD**	192.16 ± 46.89	196.91 ± 44.39	0.723
**LDL cholesterol (mg/dL), mean ± SD**	111.04 ± 41.21	122.36 ± 37.86	0.331
**HDL cholesterol (mg/dL), mean ± SD**	47.16 ± 26.13	52.91 ± 13.66	0.342
**Triglycerides (mg/dL), mean ± SD**	185.00 ± 108.35	111.73 ± 53.99	0.235
**Creatinine clearance (mL/min/1.73 m^2^), mean ± SD**	81.00 ± 15.69	91.46 ± 18.78	**0.045**

BSA, body surface area; DBP, diastolic blood pressure; DM, diabetes mellitus; HDL cholesterol, High-Density Lipoprotein cholesterol; LDL cholesterol, Low-Density Lipoprotein cholesterol; NT-proBNP, N-terminal pro B-type natriuretic peptide; SBP, systolic blood pressure; SD, standard deviation.

**Table 2:** Echocardiographic characteristics at baseline.

Variable	Patients with DM (n = 25 Patients)	Patients Without DM (n = 22 Patients)	*p*-Value
**Demographic characteristics and cardiovascular risk factors**			
**LVEDV (mL)**	110.68 ± 36.47	107.05 ± 32.20	0.718
**LVESV (mL)**	51.72 ± 20.25	47.95 ± 16.11	0.481
**LVEF (%)**	54.04 ± 5.79	54.86 ± 4.71	0.593
**E/A**	0.96 ± 0.39	1.07 ± 0.37	0.304
**E/E′ septal**	8.96 ± 2.19	8.16 ± 2.97	0.306
**E/E′ lateral**	7.48 ± 2.79	6.96 ± 2.57	0.510
**GLS (%)**	−15.18 ± 3.66	−16.89 ± 4.34	0.156
**LAVI (mL)**	60.56 ± 17.59	53.45 ± 14.52	0.136
**LASr (%)**	20.40 ± 7.25	26.68 ± 8.00	**0.007**
**LAScd (%)**	−10.92 ± 5.42	−15.64 ± 6.46	**0.010**
**LASbp (5)**	−9.92 ± 4.20	−12.87 ± 4.97	**0.034**

DM, diabetes mellitus; GLS, Global Longitudinal Strain; LASbp, left atrial strain at booster-pump phase; LAScd, left atrial strain at conduit; LASr, left atrial strain at reservoir; LAVI, Left Atrial Indexed Volume; LVEDV, Left Ventricular End-Diastolic Volume; LVEF, Left Ventricular Ejection Fraction; LVESV, Left Ventricular End-Systolic Volume.

## Data Availability

The data presented in this study are available on request from the corresponding author due to privacy.

## Supplementary Materials

The following supporting information can be downloaded at: https://www.mdpi.com/article/10.3390/jcdd12090369/s1, Table S1: STROBE Statement—adapted checklist of items to be included in reports of observational studies.

## Abbreviations

The following abbreviations are used in this manuscript:

LA	left atrium
T2DM	type 2 diabetes mellitus
LASr	left atrial strain at reservoir
LAScd	left atrial strain at conduit
LASbp	left atrial strain at booster-pump phase
HbA1c	glycosylated hemoglobin
DM	diabetes mellitus
CVD	cardiovascular disease
LV	left ventricular
LAVI	left atrial indexed volume
LAS	left atrium strain
ESC	European Society of Cardiology
CCTA	coronary computed tomography angiography
BMI	body mass index
BSA	body surface area
LDL	low-density lipoprotein
HDL	high-density lipoprotein
NT-proBNP	N-terminal proB-type natriuretic peptide
2D	two-dimensional
ASE/EACVI	American Society of Echocardiography/European Association of Cardiovascular Imaging
SD	standard deviations
ROC	receiver operating characteristic
AUC	area under the curve
SGLT2	sodium-glucose cotransporter-2
GLP-1	glucagon-like peptide-1
DBP	diastolic blood pressure
SBP	systolic blood pressure
LVEF	left ventricular ejection fraction
LVEDV	left ventricular end-diastolic volume
LVESV	left ventricular end-systolic volume
GLS	global longitudinal strain
OR	odds ratio
AF	atrial fibrillation
